# Selection for Robustness in Mutagenized RNA Viruses

**DOI:** 10.1371/journal.pgen.0030093

**Published:** 2007-06-15

**Authors:** Rafael Sanjuán, José M Cuevas, Victoria Furió, Edward C Holmes, Andrés Moya

**Affiliations:** 1 Institut Cavanilles de Biodiversitat i Biologia Evolutiva, Universitat de València, València, Spain; 2 Departament de Genètica, Universitat de València, València, Spain; 3 Instituto de Biología Molecular y Celular de Plantas, Consejo Superior de Investigaciones Científicas, Universidad Politécnica de València, València, Spain; 4 Department of Zoology, University of Oxford, Oxford, United Kingdom; 5 Center for Infectious Disease Dynamics, Department of Biology, The Pennsylvania State University, University Park, Pennsylvania, United States of America; 6 Fogarty International Center, National Institutes of Health, Bethesda, Maryland, United States of America; Fred Hutchinson Cancer Research Center, United States of America

## Abstract

Mutational robustness is defined as the constancy of a phenotype in the face of deleterious mutations. Whether robustness can be directly favored by natural selection remains controversial. Theory and in silico experiments predict that, at high mutation rates, slow-replicating genotypes can potentially outcompete faster counterparts if they benefit from a higher robustness. Here, we experimentally validate this hypothesis, dubbed the “survival of the flattest,” using two populations of the vesicular stomatitis RNA virus. Characterization of fitness distributions and genetic variability indicated that one population showed a higher replication rate, whereas the other was more robust to mutation. The faster replicator outgrew its robust counterpart in standard competition assays, but the outcome was reversed in the presence of chemical mutagens. These results show that selection can directly favor mutational robustness and reveal a novel viral resistance mechanism against treatment by lethal mutagenesis.

## Author Summary

Understanding the conditions that favor the constancy of phenotypes in the face of deleterious mutation pressure—mutational robustness—is an outstanding question in evolutionary biology. Theoretical and in silico studies utilizing digital organisms predict that slow-replicating populations can outcompete those with higher individual fitness if the former show greater robustness. This “survival of the flattest” hypothesis sits in contrast to most models of natural selection based on individual fitness, and hence challenges the “survival of the fittest” paradigm. In this work, the authors use experimental populations of the rapidly evolving vesicular stomatitis RNA virus to provide the first evidence of natural selection for mutational robustness. Based on the analysis of fitness distributions, genetic variability, and the ability to tolerate mutation accumulation, two populations with different levels of robustness were characterized. At artificially enhanced mutation rates following the application of mutagens, the more robust viral population outcompeted the other population despite having a lower replication rate. This study has important implications for lethal mutagenesis—an antiviral strategy that consists of increasing viral mutation rates through the use of mutagenic drugs—since selectively favored mutational robustness may allow RNA viruses to evolve resistance to this form of treatment.

## Introduction

Lethal mutagenesis consists of overwhelming viral populations with an excessive number of deleterious mutations and has been proposed as a candidate therapeutic strategy against RNA viruses [1–4]. Several mutagens have been used to artificially increase error rates in RNA viruses such as vesicular stomatitis virus (VSV), poliovirus type 1, foot-and-mouth disease virus, lymphocytic choriomeningitis virus, hepatitis C virus, and the human immunodeficiency virus type 1 [1]. These experiments show that while mutagens efficiently reduce viral fitness, they also impose a strong selective pressure for the evolution of resistance mechanisms. One mechanism of resistance is increased replication fidelity, which has been demonstrated in experimental populations of poliovirus type 1 subjected to ribavirin treatment [5]. Resistance mechanisms involving changes in viral polymerases that specifically reduce the incorporation efficiency of the mutagen have also been reported [6]. Another potential mechanism of resistance is increased mutational robustness, although the later remains unexplored experimentally.

Robustness to mutation determines the phenotypic expression of genetic variation and thus should be central to many evolutionary processes [7]. Insofar as it is heritable, exhibits variability among individuals, and affects the probability of survival, robustness is a potential target for selection and evolutionary optimization. However, whether robustness can be directly favored by natural selection remains controversial [8–10]. Population genetics theory predicts that robustness can only be efficiently selected for if mutation is highly frequent [7,11,12] . Similarly, quasispecies models predict that, in small replicons, robustness can significantly influence the mean fitness of the population at high mutation rates or low population sizes [13,14].

**Figure 1:**
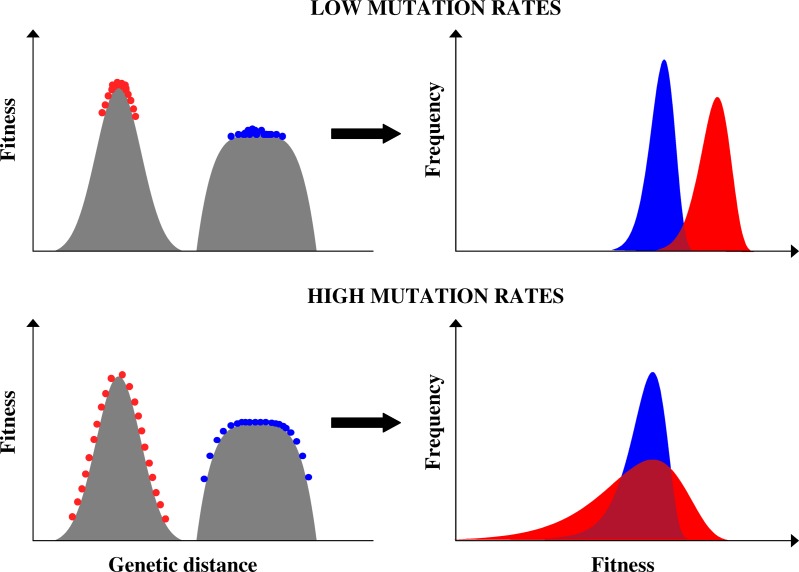
Hypothetical Populations Located at Different Regions of Sequence Space In red, a population with high replication rate but low mutational robustness. In blue, a “flat” population with lower replication rate but a higher robustness. Left panel: Dots depict individuals located on each peak at low and high mutation rates. Right panel: Qualitatively expected distribution of individual fitness values for the two populations. Individuals located at the top of the high narrow peak benefit from the highest fitness, although the other population shows lower variance in fitness due to its higher mutational robustness. At low mutation rates, the fitter population will always outcompete the flatter population, but the situation can be reversed at high mutation rates.

The conditions for the evolution of robustness have been experimentally explored in silico [15–17]. Such experiments have produced digital organisms with increased mutational robustness, although they typically pay the cost of reduced replication rates, an evolutionary trade-off that is expected from both theoretical and molecular considerations [13,18]. Specifically, following Wright's adaptive landscape model, organisms with high robustness are located in a low and flat peak, as opposed to faster but less robust replicators. Although the flatter populations should be readily outcompeted by their faster counterparts at low mutation rates, they can have a selective advantage at high mutation rates, a phenomenon dubbed the “survival of the flattest” (Figure 1) [17]. Recent experiments with viroids (plant pathogens constituted of small noncoding RNA) have revealed that a slow-replicating but highly variable species can improve its fitness relative to a faster but less variable replicator in UVC-irradiated plants, compatible with the survival of the flattest hypothesis [19].

Due to their high spontaneous mutation rates [20], RNA viruses are obvious candidates for studying the beneficial effects of mutational robustness. However, this task has to date remained elusive. Early work with the RNA phage φ6 showed that the evolution of different genotypes depended on the topology of the neighboring adaptive landscape [21]. More recent experiments with φ6 have provided some indirect evidence that robustness is an evolvable character [22]. However, direct proof of the survival of the flattest requires at least the following conditions: (i) that the outcome of competition experiments between genotypes should depend on the mutation rate and, (ii) that average mutational effects should be estimated for each competitor.

Herein, we provide evidence for the survival of the flattest using experimental populations of VSV, a lytic negative-stranded RNA virus of the *Rhabdoviridae* family. We characterized two populations adapted to a common environment but with different evolutionary histories. We studied the genetic variability and the individual fitness distribution of each population near the mutation-selection balance, and we estimated mutation rates and selection coefficients for deleterious mutations. This allowed us to conclude that one population was replicating near a high fitness peak, but was also less robust to mutation than the other. Whereas at spontaneous mutation rates, the fitter population was the best competitor, the addition of mutagens provided an advantage to the more robust, flatter, population.

## Results

### Characterization of Two Populations with Different Mutational Robustness

Two VSV populations with different evolutionary histories were chosen to start the experiments. Population A came from transfection of a full-length infectious cDNA into standard baby hamster kidney cells (BHK_21_) [23]. This cDNA was an artificial assemblage of different viral isolates and had previously experienced no propagation in natural or laboratory conditions. In contrast, population B had a complex laboratory passage history, since it originally came from a natural isolate of the Indiana serotype and, after a few passages in BHK_21_ cells, it had been replicating in human cervical cancer HeLa cells [24]. The consensus sequence of these two populations differed at 54 nucleotide positions spread throughout the genome.

A single clone from each of populations A and B was picked and evolved separately by serial passages for approximately 100 generations in the same, constant, environment, after which the two evolved lineages were assayed for fitness by standard growth assays. In accordance with previous studies [25], population A readily adapted to the new environment, showing a 53.9% fitness increase between generations 5 and 100 (Mann-Whitney test, *p <* 0.001). In contrast, population B showed a non-significant 7.0% increase in fitness (*p =* 0.075) during the same time interval. Comparison of ancestral and evolved genomic consensus sequences revealed a single fixed nucleotide substitution for population A and no changes for population B. EMBL accession numbers of the genomic consensus sequences for A and B evolved populations can be found in the Accession Numbers list of the Supporting Information section of this paper. The two adapted populations were used for all subsequent experiments. In both cases, a mutation-selection balance had probably been reached at different local peaks of the adaptive landscape, as suggested by the fact that evolution for an additional 50 generations produced no changes in mean fitness.

**Figure 2:**
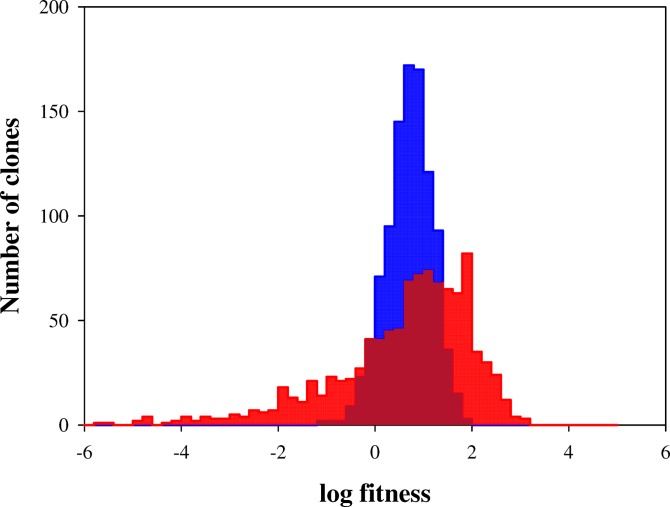
Observed Distribution of 1,000 Fitness Values for Populations A and B Based on Plaque Sizes Population A is shown in red and population B in blue. Mean log-fitness was 0.386 for population A, with variance 2.054. For population B, mean log-fitness was 0.498, with variance 0.225. Means were significantly different according to a Mann-Whitney test (*p <* 0.001). A Kolmogorov-Smirnov test also showed that the two distributions were significantly different (*p <* 0.001).

To gain some insights into the topology of each local adaptive peak, we first measured the fitness of 12 randomly chosen clones from both A and B. Although there were no significant differences in average fitness (Mann-Whitney test, *p =* 0.143), it was striking that fitness variance among clones was 30 times higher for population A than for population B. We therefore investigated each population more thoroughly. Because of the large samples required, we used lysis plaque size as a proxy to measure fitness rather than standard growth assays. Preliminary experiments with 12 clones from each population showed that fitness and log-plaque size were highly correlated (Pearson *r =* 0.944 for A, *r =* 0.959 for B, *p <* 0.001 in both cases). Estimation of fitness values of 1,000 random individual clones from each population showed that population A contained the fittest variants but also a large tail of unfit clones, whereas the fitness distribution of population B fitness was more tightly clustered around intermediate values (Figure 2). Reduced fitness variance in population B could be due to either a lower mutation rate or a higher mutational robustness.

We further characterized each population by studying their genetic heterogeneity at two variable regions of the genome. Region P covered half of the gene encoding the viral phosphoprotein, a short intergenic region, and a small fraction of the gene encoding the matrix protein, whereas region G was entirely located within the viral envelope glycoprotein gene. After obtaining cDNAs by reverse transcription of purified RNAs, these regions were amplified by PCR, cloned, and sequenced. In population A, six different mutations were found in 87 clones, giving a mutation frequency of 1.35 × 10^−4^, whereas in population B, this frequency was elevated to 2.91× 10^−4^ (Table 1). The observation that population B was roughly twice as variable as A can be explained by a higher mutation rate, but also by increased robustness. Recalling that the characterization of individual fitness distributions (Figure 2) indicated that population B should have either a lower mutation rate or increased robustness, that B has increased robustness seems to be the only hypothesis compatible with the observations.

**Table 1:**
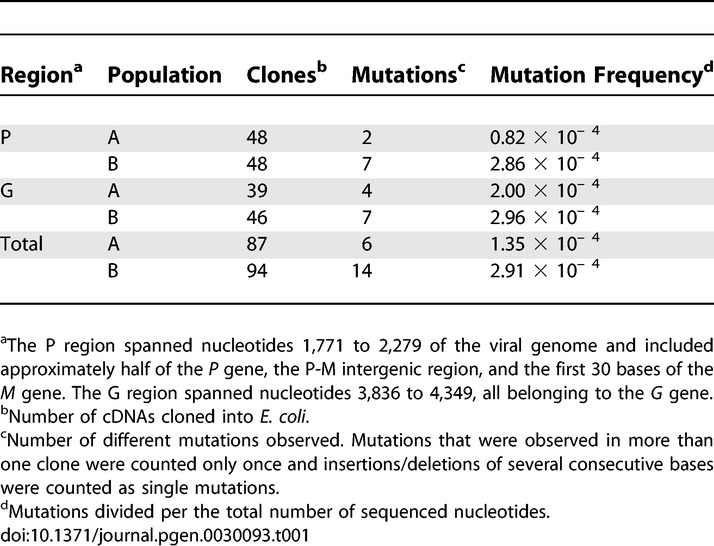
Genetic Variability of VSV Populations A and B

### Competition Assays in the Presence or Absence of Chemical Mutagenesis

**Figure 3:**
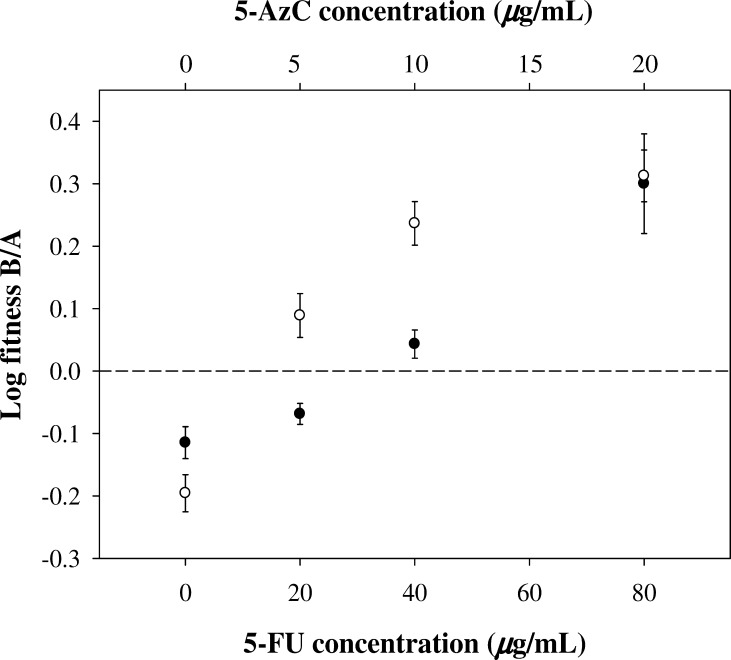
Competition Experiments at Varying 5-FU and 5-AzC Doses Open circles and filled circles represent 5-FU and 5-AzC values, respectively. The log-fitness of population B relative to population A, log(*W_B_*/*W_A_*), is represented. Bars indicate 95% confidence intervals.

If B was indeed more robust to mutation than A, we expected that their relative fitness should depend on the mutation rate. To test this prediction, we performed competition experiments taking advantage of the fact that the two populations were easily distinguishable by a monoclonal antibody resistance marker situated on genotype B. Standard assays with a 1:1 input ratio indicated that population A had an advantage over B (log relative fitness, log*W_B_*_/*A*_ = −0.114 ± 0.012, Mann-Whitney test: *p =* 0.002), confirming that A was located at a higher fitness peak. We then carried out the same experiments in the presence of the mutagen 5-fluorouracyl (5-FU). As expected, the outcome of the competition steadily changed for increasing concentrations of 5-FU (Spearman *ρ =* 0.933, *p <* 0.001) and B prevailed beyond a dose of ~30 μg/mL (Figure 3). To assess whether this pattern was drug specific, we repeated the competition assays using 5-azacytidine (5-AzC) instead of 5-FU. Similarly, we observed that B improved its performance relative to A as the dose of 5-AzC increased (Spearman *ρ =* 0.854, *p <* 0.001; Figure 3).

If selection was insensitive to mutation and hence targeted individual clones, we expected that the faster replicators of population A would always outcompete those of population B. A thousand simulations in which the empirical distributions of individual fitness values were used to predict the outcome of the competition confirmed that, if selection acted on individual clones, we should expect A to be the winner of the competition. Whereas the results from real competition experiments in the absence of mutagens were consistent with this prediction, competitions with mutagens were the opposite, suggesting that selection was favoring the population with the higher average growth rate, even though it did not harbor the fittest individuals. The most likely scenario is thus that population B was located at a lower but flatter peak of the fitness landscape, in which mutations would tend to be less deleterious.

To exclude the possibility that the RNA polymerase encoded by genotype B had an increased ability to selectively purge base analogs from its active site, we obtained molecular clone sequences of the P and G regions after three passages at 80 μg/mL 5-FU. As expected, the presence of 5-FU altered mutational patterns and increased mutation frequencies. Whereas 44% of all spontaneous nucleotide substitutions were transitions, after 5-FU mutagenesis, this percentage increased to 90%. Mutation frequencies increased to 7.09 × 10^−4^ for population A and to 9.55 × 10^−4^ for population B (Table 2). Compared to the spontaneous mutation rate (Table 1), the relative increase in mutation frequency was higher for population A, whereas in absolute terms, it was higher for B. However, population B remained more variable than A after 5-FU mutagenesis, demonstrating that even if there were differences in the ability to exclude 5-FU, these could not account for the observations. Similarly, it is in principle possible that genotype B might have a better ability to replicate in cells stressed by mutagenesis, although this explanation is similarly unable to account for all observations.

**Table 2:**
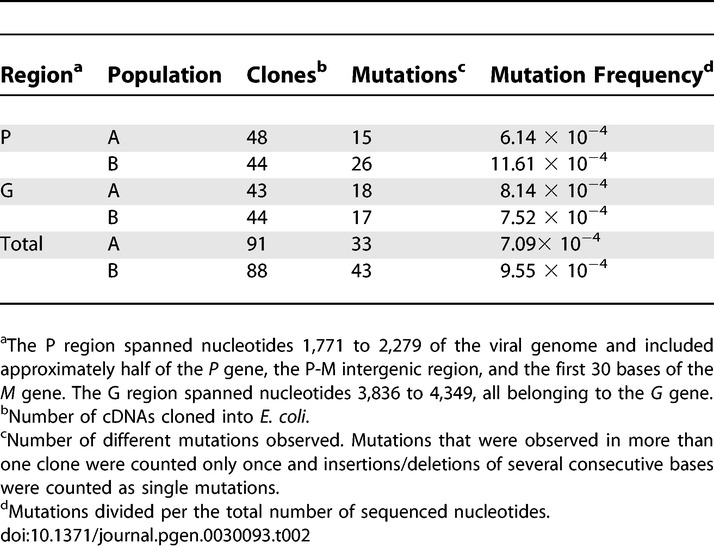
Genetic Variability of the VSV Populations A and B in the Presence of the Mutagenic Base Analog 5-FU

**Figure 4:**
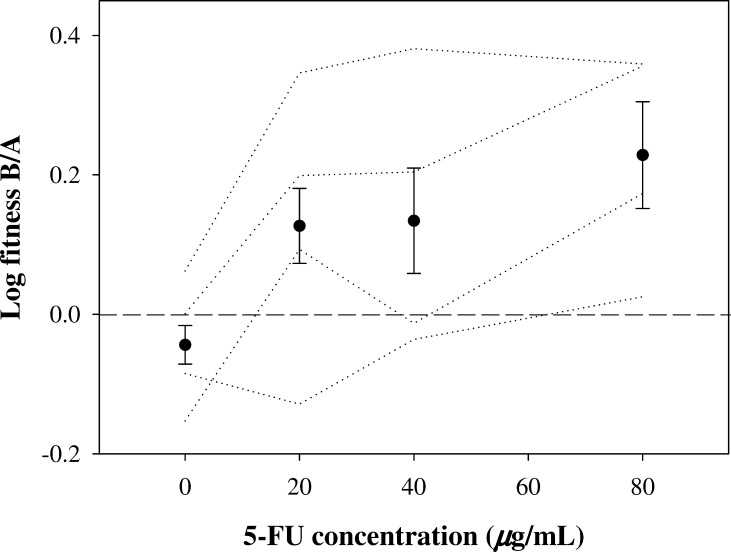
Competition Experiments between Pairs of Clones at Varying 5-FU Doses The log-fitness of clones from population B relative to those from A, log(*W_B_*/*W_A_*), is represented. Dotted lines represent each of the four clone pairs, circles the grand-log-fitness mean, and bars indicate standard errors of grand means. An analysis of the variance revealed a significant effect of both the clone pair (*F_3,43_ =* 9.871, *p <* 0.001) and the 5-FU dose (*F_1,43_ =* 9.871, *p =* 0.001) on the observed mean log-fitness.

The conclusion that B is more robust to mutation than A relies on the assumption that no beneficial mutations were sweeping through either population at the time of competition assays. Several precautions were taken to ensure that this requisite was met. First, as mentioned above, experimental evolution for additional 50 generations produced no changes in mean fitness, suggesting that beneficial mutations were not frequent. Second, competition assays were seeded with only ~100 plaque-forming units (pfu) of each population to minimize the probability that a rare beneficial mutation distorted the results. Using binomial probabilities, it can be proven that high fitness variants were very unlikely to be present in the inoculum of the competitions experiments and at the same time, go unnoticed during the plaque size fitness screening. Third, we repeated the above competition experiments using individual clones instead of populations. To do that, we randomly selected four clones from each population and we did one-to-one competitions against each of four clones drawn from the other population. We observed a significant correlation between the 5-FU dose and the log-fitness of B clones relative to A clones (Spearman *ρ =* 0.406, *p =* 0.004), making it apparent that, on average, competitions between single clones reproduced the same trend observed with populations (Figure 4).

### Mutation Accumulation under Genetic Drift

**Figure 5:**
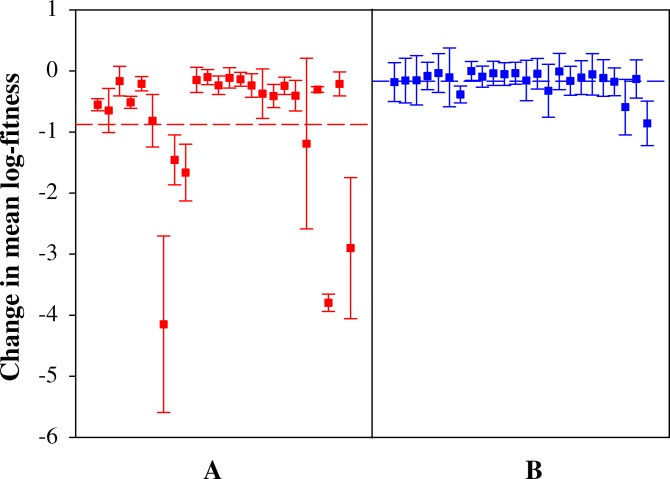
Change in Mean Log-Fitness in Mutation Accumulation Lines Derived from Populations A and B For A and B, each of the 24 lines is shown. Bars indicate 95% confidence intervals. Horizontal lines indicate the grand mean change in log-fitness.

Serial passages were done at the lowest possible population size to minimize the action of natural selection and hence favor the fixation of deleterious mutations via genetic drift. If it is true that genotype B is more robust than A, we expected this passage regime to produce a lower fitness decline in the former. For each population, starting from a single clone, we seeded 24 independent lineages that were propagated plaque to plaque for approximately 25 generations. We observed that the average fitness of these lineages decreased and fitness variance increased in both cases. However, as expected, the fitness loss was more marked for genotype A (Figure 5; Mann-Whitney test: *p <* 0.001). Similarly, the observed increase in genetic variance was much more evident for genotype A (Δlog*W_A_* = 0.876, Δlog*W_B_* = 0.169).

The expected change in average log fitness equals the product of the deleterious mutation rate (*U_d_*) and the average selection coefficient against deleterious mutations (*s*) [26,27]. Therefore, mutational robustness can be achieved by increasing the fraction of neutral mutations or by decreasing deleterious mutational effects. Although distinguishing between these two alternatives is not straightforward, mutation accumulation data can be used to estimate *U_d_* and *s* separately using the Bateman-Mukai method [26,27]. No statistical differences were observed between the two *U_d_* estimates (*U_d A_ =* 0.062, *U_d B_ =* 0.112, bootstrap test *p =* 0.247), whereas the *s* value was significantly higher for A (*s_A_* = 0.443, *s_B_* = 0.061, *p =* 0.003), suggesting that B was more robust to mutation than A because deleterious mutational effects were lower on average. We also applied a maximum likelihood method to estimate *U_d_* and to characterize the distribution of mutational effects [28], but this approach yielded nonconvergent estimates.

## Discussion

In evolutionary theory, adaptation is often assumed to be a necessarily uphill path towards the highest-fitness genotype, a paradigm known as the “survival of the fittest” [29]. However, the fittest genotype cannot be always defined in terms of individual fitness. Previous work with the RNA phage Φ6 showed that the long-term fitness of a genotype can be determined by its mutational neighborhood [21]. Here, we have characterized two VSV populations with different levels of mutational robustness and shown that this difference has a selective value at high mutation rates. Our conclusion that one population is more robust than the other is supported by the observation of three expected consequences of robustness: reduced dispersion of the individual fitness distribution, increased genetic heterogeneity, and reduced sensitivity to mutation accumulation.

In contrast to the survival of the fittest, the survival of the flattest hypothesis states that at high mutation rates, fast replicators can be outcompeted by slower replicators provided the latter benefit from increased mutational robustness. The survival of the flattest takes place when the average mutational effects influences the fitness of the population, implying that the fitness of a given genotype is determined by the occurrence of subsequent mutations or by the influx of mutations regenerating the genotype from neighbors in sequence space. These second-order effects, also termed quasispecies effects [30], are negligible for low mutation rates, but become increasingly important for higher mutation rates. Similarly, these effects become more relevant at low population sizes, because this regime favors the accumulation of mutations in the population [13]. Quasispecies effects are not fully accounted for in some classical population genetics models, which, despite incorporating the negative impact of mutation, assume that it is rare enough to allow for the neglect of multiple and back mutations. A classical formulation for asexual replicators is *W̄*= 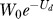 , where *W̄*is the average fitness of the population and *W*_0_ is the fitness of the fittest genotype [31]. As a consequence, the success of a given genotype can be influenced by the rate of deleterious mutation, but not by the average selection coefficient against deleterious mutations. In our experiments, *W*_0*A*_ *> W*_0*B*_ and 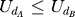 , but at artificially increased mutation rates, *W̄_A_* < *W̄_B_*, which contradicts this model and clarifies the need to consider mutational robustness to predict the fitness of viral populations subjected to mutagenesis.

RNA viruses are highly sensitive to mutation when compared to more complex microorganisms [32], a lack of robustness that is generally expected in small, compact genomes with little redundancy, no repair systems, and strong pleiotropy [13]. These species typically exist as very large populations, thus making selection efficient at purging deleterious mutations and promoting the preservation of the unmutated genotype. Genetic hypersensitivity seems to be the rule among RNA viruses but, under some conditions, the evolution of mutational robustness can be favored. Here, at the highest tested doses, 5-FU reduced the maximum viral titers by three orders of magnitude and produced more than a 3-fold increase in mutation frequencies, which should provide a favorable scenario for the evolution of robustness. Similar scenarios are likely to apply in nature following antiviral treatments [1], transmission bottlenecks [33], or host-induced mutagenesis [34].

Lethal mutagenesis has been proposed as a therapeutic strategy against RNA viruses [1–4]. For example, mutagenesis is one of the mechanisms of action of ribavirin, a drug that is currently used in combination with interferon to combat hepatitis C [35]. However, RNA viruses are known to have a remarkable potential for escaping antiviral strategies and evolving resistances. Previous work has shown that poliovirus-1 and foot-and-mouth disease virus can increase their replication fidelity or substrate specificity in response to ribavirin treatment [5,6]. By showing that robustness is a selectable trait at high mutation rates, our results reveal a novel resistance mechanism against lethal mutagenesis and open several research avenues. For example, the genetic basis of increased robustness in genotype B still needs to be identified. Here, 53 substitutions separate the two genotypes, but thermodynamic analyses predict that one or few changes suffice to modify the robustness of a given protein [36]. Related to this issue, it remains unclear whether the transition from a non-robust to a robust state can occur as a direct consequence of the selective pressure exerted or, in contrast, an episode of genetic drift is required to produce a jump in the adaptive landscape. Finally, adaptation to mutagenesis also has consequences for the evolvability of RNA viruses. Whereas the evolution of increased replication fidelity has been shown to restrict RNA virus evolvability [37], increased robustness may favor the generation of inconspicuous genetic variation and foster long-term evolvability [7,38].

## Materials and Methods

### Viruses and cells.

BHK_21_ (American Type Culture Collection, http://www.atcc.org) were cultivated at 37 °C, 5% CO_2_, in 100 cm^2^ plates under 12 mL of Dulbecco modified Eagle's minimum essential medium (DMEM) supplemented with 10% fetal calf serum, and passaged upon confluence. The VSV infectious cDNA used to obtain population A was kindly provided by G. T. W. Wertz, University of Alabama, United States of America [23].

### Viral titrations.

A 100-μL volume of an appropriate dilution was added to 60 mm culture plates and cells were topped with DMEM medium containing 0.4% agarose. Monolayers were stained at 18–22 h post-inoculation (hpi) with a solution of 2% crystal violet (Sigma, http://www.sigmaaldrich.com) in 10% formaldehyde (Panreac, http://www.panreac.com/new/ing/menu.htm).

### Viral adaptation to a constant environment.

Approximately *N*_0_ *=* 5 × 10^3^ pfu were inoculated to *C =* 10^6^ BHK_21_ cells in 25 cm^2^ flasks, incubated for 24 h, and the supernatant was used to seed a new infection passage under the same conditions, up to 25 passages. Viral population sizes at the end of each infection passages were approximately *N_f_ =* 5 × 10^9^ pfu. The number of viral infectious cycles (generations) per passage was estimated from *N*_0_, *N_f_*, and *C* as previously described [39], giving ~4 generations per passage.

### Consensus sequencing.

Viral RNA was extracted from the supernatant of the infected cultures using the High Pure Viral Nucleic Acid Kit following the manufacturer instructions (Roche, http://www.roche.com). The anti-genomic VSV cDNA was synthesized using the reverse transcriptase of the Moloney murine leukemia virus (Promega, http://www.promega.com) plus a battery of random hexamers (Promega). The genomic region containing the original mutations was amplified by PCR using *Taq* polymerase (Amersham, http://www.gehealthcare.com) and specific primers. Sequencing was carried out using ABI PRISM BigDye Terminator v3.0 Ready Reaction Cycle Sequencing KIT (Applied Biosystems, http://www.appliedbiosystems.com) on an ABI 3700 automated sequencer. Sequences were visualized and edited with the Staden software package (http://staden.sourceforge.net).

### Molecular clone sequencing.

RNA viral extraction, RT-PCRs, sequencing, and editing of sequences were performed as described above. A first round of PCR was carried out using Pfu polymerase (Amersham) and specific primers [40]. Amplified DNA products of each region were purified with High Pure PCR product Purification Kit (Roche) and directly cloned into EcoRV-digested pBluescript II SK (+) phagemid (Stratagene, http://www.stratagene.com). A second round of PCR was then carried out by adding a single transformed bacterial colony in each PCR tube and using Taq polymerase. Vector-based primers KS and SK (Stratagene) were used in this second round of PCR, as well as for the subsequent sequencing. Before sequencing, amplified DNA was purified using a PCR clean-up kit (Macherey-Nagel, https://www.macherey-nagel.com). Plasmid DNA was purified with High Pure Plasmid Isolation Kit (Roche) and clones were sequenced using vector-based primers KS and SK (Stratagene). EMBL accession numbers of the obtained sequences for nonmutagenized populations and for 5-FU mutagenized populations are in the Accession Numbers list in the Supporting Information section of this paper.

### Fitness assays.

Following protocols established in previous work [41–43], we used growth rate assays to estimate fitness. We seeded ~5 × 10^3^ pfu of each population into ~10^5^ BHK_21_ cells and incubated the culture until the population grew up to a titer of ~10^7^ pfu/mL (i.e., 7–8 hpi). From final and initial titers, the growth rate (*r*) was calculated as the slope of log-titer regression against time (hpi). We defined fitness (*W*) as the number of descendants per individual per hour, i.e., *W = e^r^* − 1. Fitness assays were done in triplicate.

### Plaque size analysis.

Viruses were plated as detailed above, paying care to put less than 50 pfu per plate to avoid plaque overlapping. All platings were done in a single block and the same overlay medium batch was used for both populations and staining was done at 24 hpi. Pictures of the plates were taken with a 5 megapixel Canon PowerShot G5 digital camera (http://www.canon.com) and image analysis was done with AnalySIS v.3.2 software (Soft Imaging System, http://www.soft-imaging.net). The vast majority of plaques were automatically identified, whereas the rest were manually delineated in a zoomed image. After defining plaques as single objects, we automatically obtained their surface area in pixels, *S*.

### Estimation of fitness from plaque sizes.

To calibrate the relationship between plaque size and fitness, we selected 12 clones from each population that widely varied in fitness and, for each, we determined the average plaque size from four independent plates. Since we had no evidence that fitness and plaque area were linearly related, we performed a log–log regression to obtain a calibration line of the form log*W = p + m*log*S*. For population A, we obtained *m =* 1.907 ± 0.318 and *p =* −13.785 ± 2.246 (*r =* 0.884), whereas for population B, we obtained *m =* 0.927 ± 0.096 and *p =* −5.874 ± 0.678 (*r =* 0.950). The calibration line was used to transform each observed plaque size into a predicted fitness.

Although the calibrations described above seemed relatively accurate, it is unavoidable that there was some degree of uncertainty and consequently no guarantee that no bias was introduced. We therefore carried out a second analysis that bypassed the calibration step. To achieve this, we simply assumed that the number of lysed cells was proportional to the number of viruses produced. First, we estimated the number of lysed cells on a plaque, *D,* by dividing its pixel surface area per the total pixel surface area of the 60 mm plate and multiplying per the total number of cells on a plate, which was obtained by counting cells in an hemocytometer (Neubauer). Second, we estimated the average number of viruses produced per cell (*K*) by titrating a fully lysed plate. This was done separately for populations A and B, yielding *K =* 2,204 ± 32 and *K =* 2,712 ± 82 respectively. Using these estimations, plaque sizes were transformed into predicted fitness as *W_pred_ =* (*KD*)^1/24^ − 1. The results obtained with this latter fitness estimation method (unpublished data) were consistent with those obtained using the calibration line (population A showed higher maximum fitness and higher variance).

### Competition assays.

Standard growth rate assays were performed as described above except that in competitions between populations (Figure 3), only 100 pfu from each competitor were inoculated to minimize the effect of rare beneficial mutations (see below). Viruses from the two genotypes were inoculated into the same well. Genotype B carried a monoclonal antibody resistance marker that allowed us to estimate the titer of each population in the mixture by plating in presence and absence of antibody. Samples were taken at 0, 7, 8, 9, 10, 11, 12, 15, 20, 25, and 30 hpi for competition with no mutagen. For competitions in the presence of 5-FU or 5-AzC, cells had been pretreated with the indicated 5-FU concentration 12 h prior infection. Samples were taken at 0, 15, 20, 25, 30, 35, 39, and 49 hpi for 20 and 40 μg/mL 5-FU and at 0, 20, 25, 30, 35, 39, and 49 hpi for 80 μg/mL 5-FU. Samples were taken at 0, 10, 12, 15, 24, 28, 32, 38, and 48 hpi for 5 and 10μg/mL 5-AzC, and at 0, 24, 28, 32, 38, and 48 hpi for 20 μg/mL 5-AzC. Growth rates for each population were calculated as the slope of the log-titer against hpi during the exponential growth phase. For competitions between populations, 12 replicates of the competition assay were done for each different mutagen dose, whereas for competitions between clones three replicates were done for each dose and each of the four clone pairs.

### Discarding rare beneficial mutations in the competition assays.

We reduced the initial inoculum to ~100 pfu of each population to minimize the effect of rare beneficial mutations in the outcome of the competition. Since 1,000 clones were sampled during the plaque size fitness screening, the probability that beneficial mutations at a frequency *f* in the population went unnoticed during this screening is given by the binomial distribution function, *Bi*(0, 1,000, *f*). For each competition assay, the probability that these beneficial mutations were present in the inoculum is 1 − *Bi*(0, 100, *f*). Therefore, for each replicate of the competition assay, the joint probability that beneficial mutations that had been missed in the plaque size screening and were present in the competition assays is *p = Bi*(0, 1,000, *f*) (1 − *Bi*(0, 100, *f*)). For all values of *f, p* ≤ 0.035 for each replica of the competition assay, thus making it very unlikely that rare beneficial mutations had affected the results of the competition after 12 replicates.

### Competition assay simulations.

The expected outcome of a competition between population A and B, based on plaque sizes, was obtained as follows. First, to mimic the inoculum size of the real competition assays, we randomly sampled a subset of 100 individuals from each population and we got their expected progeny per hour, which is equal to the predicted fitness. We then let the simulated growth proceed until the total population size was similar to that observed at the last time point of real competitions. The expected winner of the competition was the one with the larger progeny number at the final time point. The random sampling and the simulation were repeated 1,000 times.

### Mutation accumulation experiment.

Two random clones from populations A and B were picked from a 60 mm plate and stored at −80 °C as ancestors. Twenty-four mutation accumulation lines were founded from each ancestor by randomly picking 24 lysis plaques. Following previous plaque-to-plaque experimental designs [44], for each lineage, viruses were plated and at 24 hpi, a lysis plaque was randomly sampled, resuspended in DMEM medium, and directly plated onto a fresh monolayer. This protocol was pursued on a daily basis for12 passages. The number of generations elapsed during each plaque-to-plaque passage was estimated to be approximately two. To obtain enough viruses for fitness assays, the ancestors and the final derived clones were given a single passage in a 96-well plate containing ~10^4^ BHK_21_. Fitness assays were performed as detailed above simultaneously for the ancestors and the derived lines in three experimental blocks. In each block, the 24 derived clones of each population were assayed once, whereas the ancestors were assayed six times.

### Bateman-Mukai method.

The expected change in log-fitness after *t* generations (*t =* 24) is Δlog*W =* log*W_t_* − log*W*_0_ *= U_d_tE*[log(1 − *s*)]. The expected genetic variance for log-fitness among lineages is σ*_G_* ^2^(log*W_t_*) ≈ *U_d_tE*[(log(1 − *s*))^2^], and the total variance is σ*_T_* ^2^(log*W*) = σ*_E_* ^2^(log*W*) + σ*_G_* ^2^(log*W*) where sub-index *E* refers to environmental variance. Since genetic variance is null for the ancestor and all fitness assays were performed in the same environmental conditions, σ*_E_* ^2^(log*W_t_*) = σ*_E_* ^2^(log*W*_0_) = σ*_T_* ^2^(log*W*_0_), and therefore σ*_G_* ^2^(log*W_t_*) = σ*_T_* ^2^(log*W_t_*) − σ*_T_* ^2^(log*W*_0_) = Δσ*_T_* ^2^(log*W*). It follows that

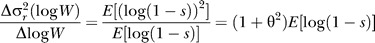
and that

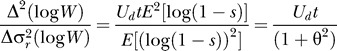
where *θ* is the coefficient of variation (standard deviation to mean ratio) of log *W* associated to single deleterious mutations. (1 + *θ* ^2^)*E*[log(1 − *s*)] and *U_d_*/(1 + *θ* ^2^) were directly estimated from the data.

For an exponential distribution (a simple and relatively accurate model for describing fitness effects associated to single mutations), *θ =* 1. Nonetheless, the actual distribution of mutational effects might have a heavier tail and thus a *θ >* 1. A previous fitness dataset of 28 nonlethal random mutants of VSV [42] gave the empirical estimation *θ =* 1.598. Using this figure, we obtained estimates of *U_d_* and *E*[log(1 − *s*)] and then, of *s*. For A, *θ =* 1.598 should be an accurate estimation of the true *θ*-value, because it was obtained for the above-mentioned full-length infectious cDNA clone. It is possible, however, that, due to its higher mutational robustness, B showed a lower *θ*-value. This potential bias could account for the slightly higher *U_d_* estimate in B, but it could not account for the nearly one order of magnitude difference in *s*-values (in the extreme case *θ_B_ =* 0, the true *U_d B_* would be 2.598 times lower than our estimate and the true *s_B_* 2.598 times higher).

To address whether differences between *U_d_* and *s* between A and B were statistically significant, we generated 1,000 bootstrap pseudo-replicates from both the 24 averaged fitness values of the derived clones and the six averaged fitness values of the ancestors. After obtaining the corresponding 1,000 pseudo-replicates of *U_d_*, we counted the number of times *U_d_* was larger for A versus B, and the same for *s*.

Parameters *U_d_* and *s* were also estimated using the maximum likelihood approach implemented in the program MLGENOMEU (http://homepages.ed.ac.uk/eang33/mlgenomeu/mlginstructions.html), in which a Gamma distribution of the form *Ga*(*s*) = α*^β^s^β^* ^− 1^ *e* − ^α*s*^/Γ(*β*) is used to describe the distribution of mutational effects (*s* =*β/α*) [28]. Following the author's recommendations, we ran the program to estimate *U_d_* and *α* at fixed *β*-values varying from 99 to 0.01. The log likelihood monotonically increased as *β* decreased, yielding increasingly higher *α*-values (*s* → ∞ and *U_d_* → 0).

### Statistics.

Statistics were done with MS Excel (http://www.microsoft.com) and the SPSS 12.0 package (http://www.spss.com). Resamplings and simulations were done using a Perl script.

## Supporting Information

### Accession numbers

The European Molecular Biology Laboratory (EMBL) (http://www.ebi.ac.uk/cgi-bin/emblfetch) database accession numbers for the A and B evolved populations are AM690336 and AM690337, respectively. For nonmutagenized populations, accession numbers are AM689332–AM689519 and for 5-FU mutagenized populations, accession numbers are AM689705–AM689876.

## Abbreviations

5-AzC: 5-Azacitydine
5-FU: 5-Fluorouracyl
BHK_21_: baby hamster kidney cells
hpi: hours post-inoculation
pfu: plaque-forming unit
VSV: vesicular stomatitis virus
